# Occult cause of uveitis-glaucoma-hyphema syndrome diagnosed during treatment with endocyclophotocoagulation (ECP)

**DOI:** 10.1016/j.ajoc.2022.101537

**Published:** 2022-04-12

**Authors:** Amol A. Sura, Amit K. Reddy, Kelly Babic, Murtaza Saifee, Nisha R. Acharya, John A. Gonzales, Ying Han, Thuy A. Doan

**Affiliations:** a Francis I. Proctor Foundation, University of California San Francisco, San Francisco, CA, USA; b Department of Ophthalmology, University of California San Francisco, San Francisco, CA, USA

**Keywords:** UGH, Hypertensive uveitis, Uveitis, Hyphema, Vitreous hemorrhage, Endocyclophotocoagulation

## Abstract

**Purpose:**

To describe uveitis-glaucoma-hyphema (UGH) syndrome secondary to a posterior chamber intraocular lens (PCIOL) within the capsular bag in which pathogenic changes to the ciliary body were observed and treated with endocyclophotocoagulation (ECP).

**Observations:**

An 85-year-old woman who had cataract surgery in her right eye four years ago presented with recurrent, unilateral, open-angle, hypertensive uveitis in her right eye. Her presentations were characterized by decreased vision, elevated intraocular pressure, corneal edema, a mixed anterior chamber reaction, and pigmented anterior vitreous cells. She had a frank vitreous hemorrhage during two episodes. Ultrasound biomicroscopy revealed a dense Soemmerring ring in her right eye without evidence of PCIOL-iris or PCIOL-ciliary body chafe. Subsequent ECP revealed whitened and atrophic ciliary processes adjacent to a tilted haptic within the capsular bag, consistent with chronic PCIOL-ciliary body chafe. ECP was applied to the affected ciliary processes, which successfully eliminated recurrences.

**Conclusions and importance:**

UGH can rarely occur due to an PCIOL within the capsular bag. In cases where ultrasound biomicroscopy (UBM) does not show abnormalities and clinical suspicion remains high, ECP can be a useful adjunct to observe and treat abnormalities of the ciliary body.

## 1 Introduction

Uveitis-glaucoma-hyphema syndrome (UGH) is an adverse outcome of cataract surgery that typically arises when a rigid intraocular lens (IOL) chafes against pigmented anterior segment structures. Historically,UGH occurred when rigid anterior chamber IOLs would chafe against the iris, resulting in its namesake clinical features of anterior segment inflammation, hyphema, and elevated intraocular pressure. Following modern cataract surgery, UGH more commonly occurs as a complication of sulcus- or posterior-chamber IOLs, especially single-piece IOLs in the ciliary sulcus or malpositioned IOLs. The clinical phenotype has also evolved. It less commonly results in hyphema, whereas common presentations now include cystoid macular edema, recurrent vitreous hemorrhage, and corneal decompensation. It can present as recurrent, unilateral, open-angle, hypertensive uveitis.^1^ Here, we describe a case of UGH syndrome due to a single-piece, posterior chamber IOL in the capsular bag. The diagnosis was made by direct visualization of the affected ciliary process using an endoscopic probe.

## 2 Case report

An 85-year-old woman presented to the Emergency Department with acute-onset blurry vision in her right eye. An outside Emergency Department recorded an intraocular pressure (IOP) of 80 mmHg. After administering 500 mg oral acetazolamide and multiple rounds of IOP-lowering drops, they emergently referred her to the ophthalmology clinic at UCSF. She had cataract surgery right eye in 2017 and left eye in 2010. She had a history of hypertension, hyperlipidemia, and shingles on her trunk.

**Fig. 1:**
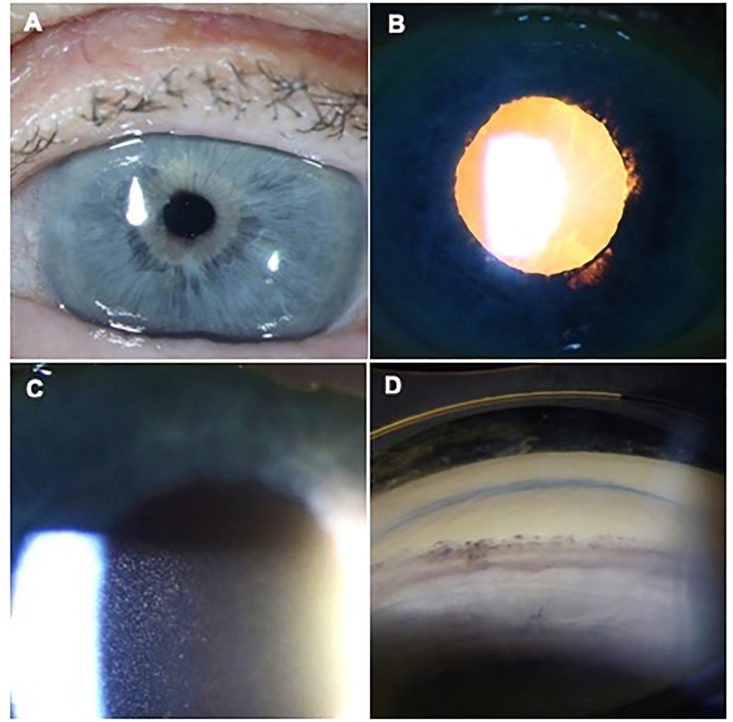
Clinical exam of the right eye. External photographs demonstrating: **A.** Irregular pupillary ruff. **B.** Peripupillary transillumination defects. **C.** Dense pigmented cellular reaction of the anterior chamber during an acute flare. **D.** Open angle and pigmented Schwalbe's line on gonioscopy.

On examination, her best-corrected visual acuity was 20/400 in the right eye and 20/20 in the left. Her IOP was 12 in the right eye and 11 in the left eye. There was no relative afferent pupillary defect. Her right eye exam (Fig. 1) demonstrated a cornea with microcystic edema and Descemet folds, anterior chamber with 4+ mixed anterior chamber cells without hypopyon or hyphema, iris with poor dilation and peripupillary transillumination defects, a posterior chamber intraocular lens (PCIOL), and anterior vitreous with 2+ pigmented cells. She did not have pseudophacodonesis. Gonioscopy and dilated fundus exam were unremarkable.

Her left eye was normal except for iris peripupillary transillumination defects and a PCIOL.

Laboratory workup that was negative or normal included: complete blood count, comprehensive metabolic panel, treponemal antibodies, QuantiFERON Gold TB, human-leukocyte antigen-B27, erythrocyte sedimentation rate, C-reactive protein, prothrombin time, partial thromboplastin time. A chest x-ray was negative. Polymerase chain reaction testing of her right eye aqueous fluid was negative for varicella zoster virus, herpes simplex virus, and cytomegalovirus. Metagenomic deep sequencing of her aqueous fluid was negative for occult infections.

She was started on topical prednisolone acetate 1% every hour and cyclopentolate 1% three times daily in her right eye. She was continued on dorzolamide 2%-timolol 0.5% two times daily and brimonidine 0.2% three times daily in the right eye. Her vision recovered to 20/20, and she was tapered off all drops.

**Fig. 2:**
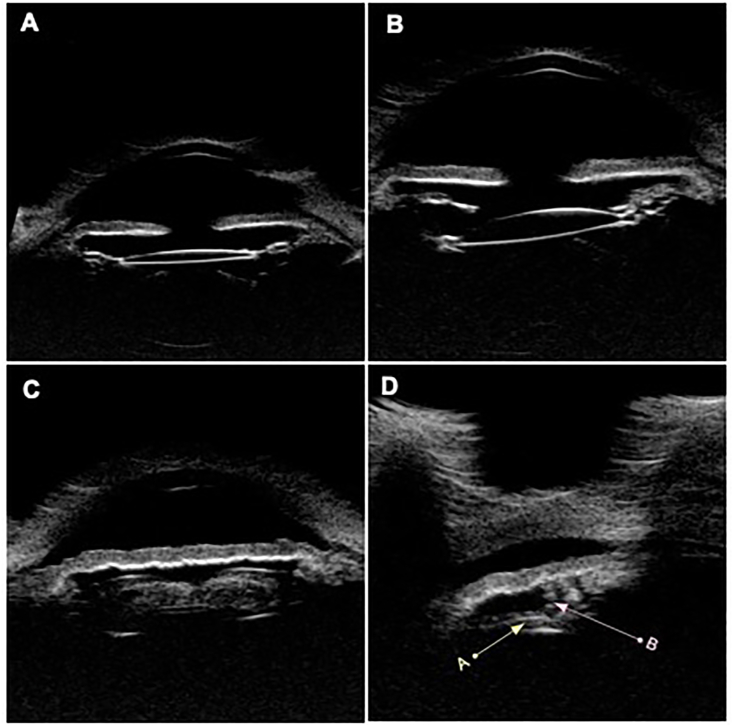
Ultrasound biomicroscopy of both eyes **A.** Horizontal axial image of the symptomatic right eye with an appropriately-positioned lens-bag complex. **B.** Horizontal axial image of the left eye with a posteriorly displaced lens-bag complex. **C.** Large amount of retained cortical material within the capsular bag, right eye. **D.** The lens haptic (yellow arrow A) appears appropriately positioned relative to the ciliary processes (pink arrow B), right eye.

Two months later, she recurred with another episode of acute-onset blurry vision, IOP of 47 mmHg, corneal edema, and 3+ mixed anterior chamber cells. Her vision and pressure promptly normalized with topical corticosteroids and aqueous suppressants. Over the subsequent three months, she recurred five additional times despite being on prophylactic topical corticosteroids and aqueous suppressants. Recurrences became more frequent and severe. Her fourth recurrence demonstrated a microhyphema. Her fifth and sixth recurrences demonstrated dense vitreous hemorrhages with decreased vision. B-scan ultrasonography and fluorescein angiography were normal. Ultrasound biomicroscopy of her anterior segment revealed dense cortical material (Soemmerring's ring) in her affected right eye. Incidentally, her unaffected left eye revealed a posteriorly displaced lens-bag complex (Fig. 2). Neither eye demonstrated evidence of lens-iris chafe or lens movement in upright and prone positions.

**Fig. 3:**
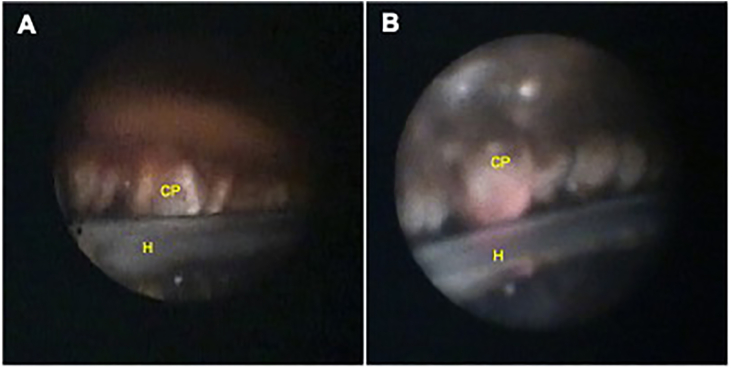
Intraoperative photographs during endocyclophotocoagulation demonstrate evidence of uveitis-glaucoma-hyphema syndrome **A.** One clock hour of nasal ciliary processes (CP) appeared whitened, atrophic, and abnormally close to the lens haptic (H), which is still within the capsular bag. **B.** Image demonstrating the ciliary process in contact with the haptic.

Due to a continued concern for mechanical lens trauma, she underwent surgical endoscopic exploration of her right eye ciliary body. An anterior approach was used to explore the ciliary sulcus above the IOL. No vitrectomy was performed. She was found to have an anteriorly displaced nasal haptic within the capsular bag adjacent to whitened and atrophic ciliary processes (Fig. 3), suggestive of chronic lens-ciliary body chafe. The lens was repositioned posteriorly, and the adjacent ciliary processes from 2:30 to 3:30 were treated with endocyclophotocoagulation until the ciliary processes were shrunk and rotated away from the haptic. At last follow up ten months after the procedure, she has not recurred.

## 3 Discussion

In-the-bag PCIOLs rarely present with UGH syndrome. Multiple mechanisms have been proposed.^2^ A dislocated IOL-bag complex may result in iris or ciliary body chafing while the haptic is in the bag. A haptic can break through the equatorial bag.^3^ Other cases describe capsular fibrosis or a Soemmerring's ring inducing asymmetric forces on the IOL.^4^ This is thought to displace the IOL haptic and induce iris or ciliary body chafe through the bag. Soemmerring's ring-induced IOL displacement is most compatible with our patient's presentation, but other mechanisms such as occult ciliary body neovascularization cannot be ruled out completely.

Another unusual feature of our patient's case is that the diagnosis required endoscopic visualization of the haptic and ciliary body. High-definition ultrasound biomicroscopy (UBM) in different head positions is considered the imaging modality of choice to diagnose UGH syndrome,^5^ but it failed to reveal the pathogenic changes in our patient, possibly due to the surrounding Sommering's ring obscuring the PCIOL. Thus, ECP was a useful adjunct for diagnosis and treatment. ECP using an anterior approach was helpful because it obviated the need for a vitrectomy and did not distort the globe. A limitation is that it requires viscoelastic in the sulcus, which might change the true position of the IOL and ciliary body processes.

UGH can acutely present many years after cataract surgery. One study found that the median time from cataract surgery to diagnosis of UGH was 7.5 years. In that study, 6/14 cases were caused by PCIOLs, and all cases demonstrated abnormalities on exam or UBM.^6^ Risk factors for delayed-onset include presence of Soemmerring's ring, capsular fibrosis, or IOL instability.^2^^,^^4^

In conclusion, UGH syndrome should remain on the differential for recurrent, unilateral, hypertensive uveitis in a pseudophakic patient. It is often a diagnosis of exclusion when examination and clear UBM evidence is lacking. Clinicians should be aware that in-the-bag and delayed-onset UGH syndrome can occur. In the absence of UBM abnormalities, endoscopic visualization and cyclophotocoagulation of the ciliary processes can be helpful for diagnosis and treatment, respectively.

## Funding

10.13039/100013202University of California San Francisco Department of Ophthalmology is supported in part by an unrestricted grant from 10.13039/100001818Research to Prevent Blindness, a 10.13039/100000053National Eye Institute Core Grant (EY06190), and That Man May See Foundation.

## Author contribution

Authorship Eligibility Statement: AAS: Conceptualization, investigation, writing-original draft. AKR: conceptualization, writing-editing.: KB: investigation, writing-editing.: MS: investigation.: NRA: conceptualization, writing-editing.: JAG: conceptualization, writing-editing.: YH: conceptualization, investigation.: TAD: conceptualization, investigation, writing-editing.

## Patient consent statement

The authors certify that the patient gave oral consent for images and other clinical information relating to her case to be reported in a medical publication. The report does not contain any personal information that could lead to identification of the patient.

## Intellectual property

We confirm that we have given due consideration to the protection of intellectual property associated with this work and that there are no impediments to publication, including the timing of publication, with respect to intellectual property. In so doing we confirm that we have followed the regulations of our institutions concerning intellectual property.

## Declaration of competing interest

The authors certify that they have No funding disclosures or conflicts of interest.
